# Predialysis and Dialysis Therapies Differently Affect Nitric Oxide Synthetic Pathway in Red Blood Cells from Uremic Patients: Focus on Peritoneal Dialysis

**DOI:** 10.3390/ijms22063049

**Published:** 2021-03-17

**Authors:** Carola Palmerini, Luca Piscitani, Giuseppina Bologna, Chiara Riganti, Paola Lanuti, Domitilla Mandatori, Lorenzo Di Liberato, Giorgia Di Fulvio, Vittorio Sirolli, Giulia Renda, Caterina Pipino, Marco Marchisio, Mario Bonomini, Assunta Pandolfi, Natalia Di Pietro

**Affiliations:** 1 Department of Medical, Oral and Biotechnological Sciences, G. d’Annunzio University Chieti-Pescara, 66100 Chieti, Italy; carola_palmerini@hotmail.it (C.P.); domitilla.mandatori@unich.it (D.M.); c.pipino@unich.it (C.P.); assunta.pandolfi@unich.it (A.P.); 2 Center for Advanced Studies and Technology-CAST (ex CeSI-MeT), G. d’Annunzio University Chieti-Pescara, 66100 Chieti, Italy; giuseppina.bologna@hotmail.it (G.B.); paola.lanuti@unich.it (P.L.); marco.marchisio@unich.it (M.M.); 3 Nephrology and Dialysis Unit, SS. Annunziata Hospital, 66100 Chieti, Italy; lucpis90@virgilio.it (L.P.); lorenzo.diliberato@asl2abruzzo.it (L.D.L.); difulvio.giorgia@gmail.com (G.D.F.); vittorio.sirolli@unich.it (V.S.); mario.bonomini@unich.it (M.B.); 4 Department of Medicine and Aging Sciences, G. d’Annunzio University Chieti-Pescara, 66100 Chieti, Italy; 5 Department of Oncology, University of Torino, 10124 Torino, Italy; chiara.riganti@unito.it; 6 Department of Neuroscience, Imaging and Clinical Sciences, G. d’Annunzio University Chieti-Pescara, 66100 Chieti, Italy; grenda@unich.it; 7 Cardiology Unit, SS. Annunziata Hospital, 66100 Chieti, Italy

**Keywords:** erythrocytes, chronic kidney disease, nitric oxide, cGMP, cardiovascular disease, MRP4, peritoneal dialysis, hemodialysis

## Abstract

Red blood cells (RBCs) have been found to synthesize and release both nitric oxide (NO) and cyclic guanosine monophosphate (cGMP), contributing to systemic NO bioavailability. These RBC functions resulted impaired in chronic kidney disease (CKD). This study aimed to evaluate whether predialysis (conservative therapy, CT) and dialysis (peritoneal dialysis, PD; hemodialysis, HD) therapies used during CKD progression may differently affect NO-synthetic pathway in RBCs. Our data demonstrated that compared to PD, although endothelial-NO-synthase activation was similarly increased, HD and CT were associated to cGMP RBCs accumulation, caused by reduced activity of cGMP membrane transporter (MRP4). In parallel, plasma cGMP levels were increased by both CT and HD and they significantly decreased after hemodialysis, suggesting that this might be caused by reduced cGMP renal clearance. As conceivable, compared to healthy subjects, plasma nitrite levels were significantly reduced by HD and CT but not in patients on PD. Additionally, the increased carotid intima-media thickness (IMT) values did not reach the significance exclusively in patients on PD. Therefore, our results show that PD might better preserve the synthetic NO-pathway in CKD-erythrocytes. Whether this translates into a reduced development of uremic vascular complications requires further investigation.

## 1. Introduction

Red blood cells (RBCs) have been considered for several years exclusively as simple carriers of gas and nutrients to tissues. Until then, the evaluable changes in RBCs were only related to their number which was frequently found to be decreased, a complication known as anemia, in conditions such as cardiovascular disease (CVD) and chronic kidney disease (CKD) [1,2]. Interestingly, advances in studies over the past decade have revealed that erythrocytes are fully involved in regulating vascular tone with an active role that has been described as “erythrocrine function” [3]. Especially, this refers to the ability of RBCs to regulate the scavenging, metabolism, transport and release of Nitric Oxide (NO), its metabolites and ATP, thus controlling the systemic bioavailability of NO and vascular tone [3,4].

Indeed, we and others have shown that RBCs form NO through nitrite reduction under hypoxia conditions and synthesize NO through an active endothelial nitric oxide synthase (eNOS) during normoxia, both contributing to the circulating NO pool [5,6,7,8]. These findings have raised the idea that alterations in such and other RBCs functions may lead to increased cardiovascular risk as already hypothesized in diabetes mellitus, coronary artery disease (CAD), hypertension and CKD [8,9,10,11,12,13].

Especially for CKD, we previously showed that in RBCs from patients with end stage renal disease (ESRD) on hemodialysis a reduced expression of eNOS was associated with a compensatory increase in its activation and consequent increase of NO production [8]. This was in line with other studies showing a marked increase of NO production in RBCs and platelets from hemodialysis patients [14,15]. In support of these evidence, some mechanisms (i.e., increased shear stress and oxidative stress) have been suggested to compensate for the lack of NO production, which can be activated in several pathological conditions [8,16,17,18,19].

However, there are also opposite findings such as those regarding RBCs from patients with CAD or diabetes where a reduced intra-erythrocyte eNOS activation has been shown [7,11,20].

Moreover, alongside the alterations regarding NO production, there are evidence on cyclic guanosine monophosphate (cGMP), a known biological effector of NO, showing its markedly increase both into RBCs and platelets from patients on hemodialysis [14,15].

In particular, our previous results proved a significant retention of cGMP into RBCs from ESRD patients that was mainly ascribed to a reduced activity of the cGMP membrane transporter (MRP4, multidrug-resistance-associated protein-4), which showed a significant increase in tyrosine nitration and in cysteine nitrosylation (NOTyr-MRP4 and NOCys-MRP4, respectively) [8,21].

Based on this evidence, it is possible to assume that erythrocytes in the uremic milieu undergo molecular alterations, which, together with other changes induced by uremia, could represent an interesting pathophysiological link with the development of impaired NO bioavailability in uremia.

However, so far, such erythrocyte changes have been studied only in ESRD patients on hemodialysis, and there is a lack of studies demonstrating whether these changes can be differently affected depending on the therapy used and considering the stages of renal disease.

In this regard it is important to point out that the current international guidelines define the CKD based on the Glomerular Filtration Rate (GFR) classifying CKD in 5 different stages: stage 1 with normal or high GFR; stage 2 mild CKD; stage 3 moderate CKD; stage 4 severe CKD; stage 5 End Stage Renal Disease (ESRD) [22].

Patients who reach the ESRD stage can no longer undergo conservative treatment (CT), but they must rely on renal replacement therapy in the form of dialysis or renal transplant [23]. The two common modalities of dialysis are hemodialysis (HD) and peritoneal dialysis (PD) which are chosen through a timely decision-making process shared between the healthcare team and patients.

Studies highlighting the better dialysis therapy that could be useful in guiding the choice of patients are quite conflicting [24,25,26,27,28]. It should be considered that dialysis specific risk factors, including uremia, and the procedure itself place patients at higher risk of CVD. Nevertheless, whether the modality of dialysis represents a risk factor for cardiovascular disease is still debated [29]. However, some studies suggest possible benefits for patients receiving PD, while others claim the hemodialysis as best treatment [27,29,30,31,32,33,34].

Therefore, as the uremic environment has been shown to impair several RBCs functions [13], studying the effects that the different therapies used in CKD could have on RBCs changes would add an important piece of knowledge to this intricate scenario.

Hence, the present study aimed to evaluate whether and to what extent NO synthetic pathway in RBCs could be affected by the different CKD treatments used, including CT (CKD stage 3 and 4), PD and HD (both CKD stage 5). For the first time, the data obtained showed that peritoneal dialysis impaired the RBCs functions to a lesser extent than other CKD therapies, proving its ability to better preserve the synthetic NO pathway in erythrocytes and potentially protect against uremic vascular complications.

## 2. Results

### 2.1. Main Clinical and Biochemical Characteristics of the Study Population

As shown in Table 1, the measured mean differences for parameters that are known to be altered in uremia, such as hemoglobin, hematocrit, serum creatinine, albumin and total proteins, were statistically significant in CKD patients compared to healthy subjects (CTRL). Moreover, also triglycerides, systolic blood pressure and C-reactive protein (CRP) levels resulted significantly higher in patients.

Additionally, we assessed the carotid intima-media thickness (IMT) that is considered an index of cardiovascular risk. Interestingly, IMT values did not reach the significance in patients on PD therapy compared to CTRL, while they were significantly greater in patients on CT and HD.

### 2.2. RBC-NOS Activation

Our previous results [8] demonstrated increased activation of eNOS in RBCs from ESRD patients on hemodialysis, although the expression of this enzyme was decreased compared to RBCs from healthy subjects.

**Figure 1:**
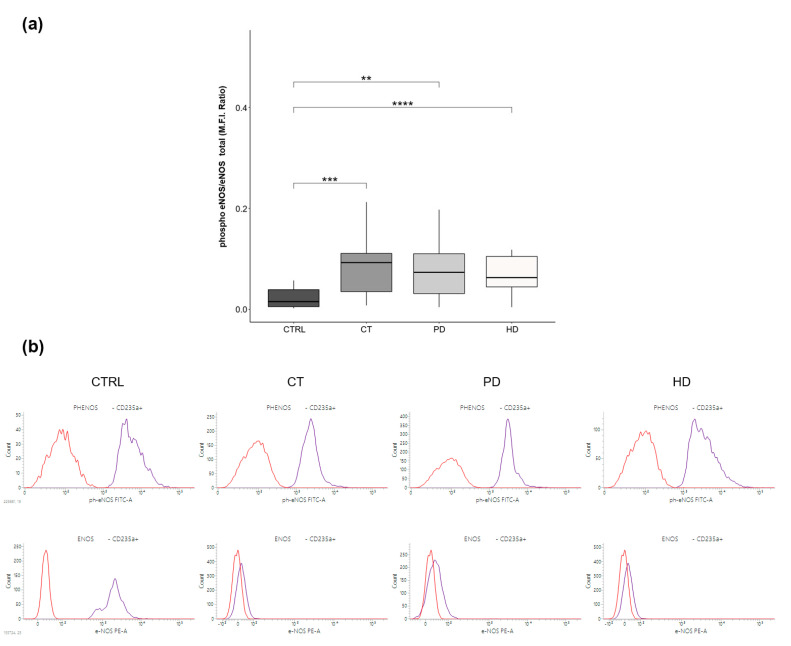
RBC-NOS phosphorylation levels. (**a**) Box plots represent eNOS phosphorylation (Ser-S1177) levels in the four study groups (CTRL, CT, PD, HD; *N* = 20 for each group). Data were obtained by flow cytometry analysis and expressed as Mean Fluorescence Intensity (MFI) Ratio of RBC ph-eNOS/eNOS. Horizontal black lines indicate the medians, boxes the interquartile range (IQR) and the bottom and top whiskers represent the minimum and maximum values. (** *p* < 0.01; *** *p* < 0.001; **** *p* < 0.0001). (**b**) The histograms representing RBCs stained by ph-eNOS (upper panel) and eNOS (lower panel) (blue)was overlaid to the histogram of the related controls (red) in four study groups (CTRL, CT, PD, HD).

Based on these findings, we assessed that Ser1177-eNOS phosphorylation levels (Figure 1) were similarly and significantly increased in RBCs from patients on CT, PD and HD compared to RBCs from CTRL subjects. This suggests a potential compensatory response induced by the uremic milieu itself and possibly independent from the type of therapy used.

### 2.3. cGMP Levels in RBCs

It has been well established from previous studies that bioavailable NO activates soluble guanylate cyclase (sGC) in RBCs inducing an increase in cGMP content [35,36]. Moreover, we previously found cGMP levels increased in RBCs from ESRD patients on HD [8].

**Figure 2:**
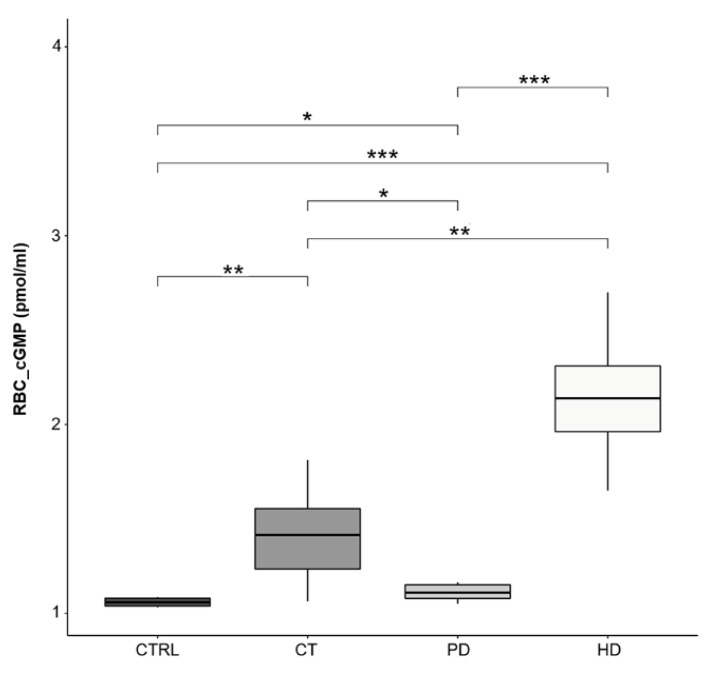
RBC cGMP levels. Box plots represent intra-erythrocytes cGMP levels in the four study groups (CTRL, CT, PD, HD; *N* = 10 for each group). cGMP concentration was evaluated through ELISA and expressed as picomoles/milliliters (pmol/mL). Horizontal black lines indicate the medians, boxes the interquartile range (IQR) and the bottom and top whiskers represent the minimum and maximum values. (* *p* < 0.05; ** *p* < 0.01; *** *p* < 0.001).

As shown in Figure 2, in this study we demonstrate that RBCs from all CKD patients showed significant increase of cGMP content compared to healthy ones. However, compared to PD, the levels of this nucleotide were significantly greater in patients on CT and HD and in particular in these patients the intra-erythrocyte cGMP level reached the highest levels.

### 2.4. MRP4 Nitration and Nitrosylation Levels in RBCs

The increased activation of RBC-NOS, described above, could itself not completely justify the increase in cGMP levels in the erythrocytes of CKD patients since significant differences in intra-erythrocyte cGMP content were found between therapies. Thus, we considered the hypothesis that the increased cGMP level may be caused by impaired extracellular efflux, as previously demonstrated [8]. To verify this, we evaluated the expression, nitration and nitrosylation levels of MRP4, a known membrane transporter of cGMP.

**Figure 3:**
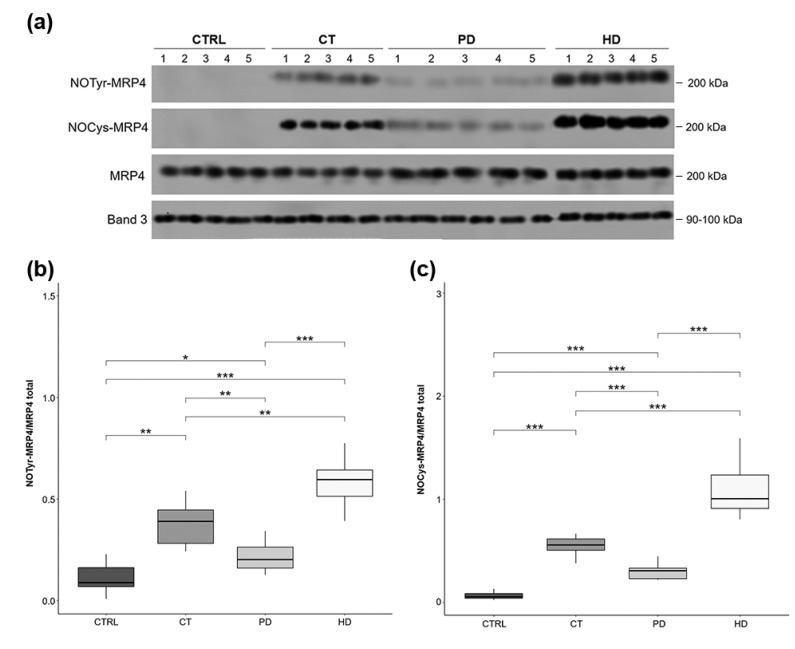
MRP4 nitration and nitrosylation levels in RBCs. (**a**) Representative Western Blot of membrane extracts obtained from all the study groups (CTRL, CT, PD, HD; *N* = 10 for each group). Band 3 was used as positive control of equal proteins loading. Box plots representing (**b**) MRP4 nitration (NOTyr-MRP4) and (**c**) nitrosylation (NOCys-MRP4) levels on MRP4 total. Horizontal black lines indicate the medians, boxes the interquartile range (IQR) and the bottom and top whiskers represent the minimum and maximum values. (* *p* < 0.05; ** *p* < 0.01; *** *p* < 0.001).

As shown in Figure 3, besides comparable constitutive MRP4 expression (Figure 3a), we found significant increased levels of MRP4 nitration (NOTyr-MRP4, Figure 3a,b) and nitrosylation (NOCys-MRP4, Figure 3a,c) in RBCs from all CKD patients compared to CTRL. Notably, compared to PD, MRP4 nitration/nitrosylation resulted significantly greater in CT and even more in HD.

Interestingly, the higher the NOTyr-MRP4 and NOCys-MRP4 levels, the greater the accumulation of cGMP (Figure 2 and Figure 3, respectively). In fact, RBCs from patients on PD who exhibited lower cGMP accumulation also exhibit weaker MRP4 nitration and nitrosylation levels than CT and HD treatments, while, as expected, HD therapy resulted the most effective.

### 2.5. MRP4-ATPase Activity in RBCs

**Figure 4:**
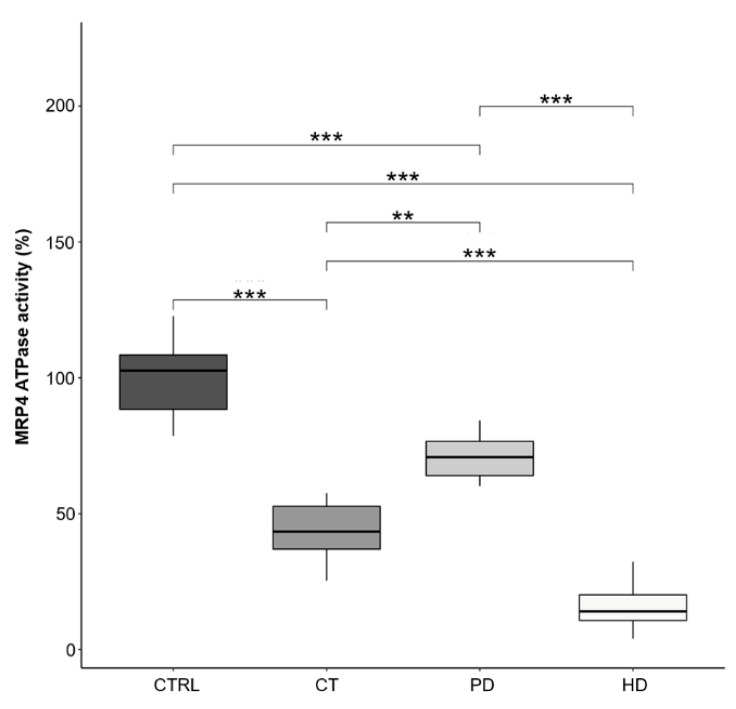
MRP4 ATPase activity in RBCs. ATPase activity was measured spectrophotometrically after immunoprecipitation of MRP4 from membrane fraction of RBCs obtained from all the study population (CTRL, CT, PD, HD; *N* = 10 for each group). Horizontal black lines in box plots indicate the medians, boxes the interquartile range (IQR) and the bottom and top whiskers represent the minimum and maximum values. (** *p* < 0.01; *** *p* < 0.001).

Subsequently, we evaluated the ATPase activity of the MRP4 transporter, which, as previously demonstrated, can be compromised following the nitration and nitrosylation process [8]. As shown in Figure 4, we demonstrated a significant decrease in the percentage of MRP4 residual activity in RBCs from HD (15.52 ± 8.30), PD (71.75 ± 7.49) and CT (43.85 ± 13.85) treated patients compared to CTRL subjects (100 ± 13.85). Interestingly, even in this case, RBCs from patients under PD therapy resulted significantly less compromised than those under CT and HD treatments.

Again, by matching the data on MRP4 activity, its nitration/nitrosylation levels (Figure 3) and cGMP accumulation (Figure 2), it appears evident that PD therapy shows better MRP4 performance in regulating erythrocytes cGMP efflux.

### 2.6. Plasma cGMP Levels

Some previous studies have shown an increased plasma cGMP in CKD patients on hemodialysis, justifying this effect as a consequence of reduced renal clearance of this nucleotide in uremia [15,37]

**Figure 5:**
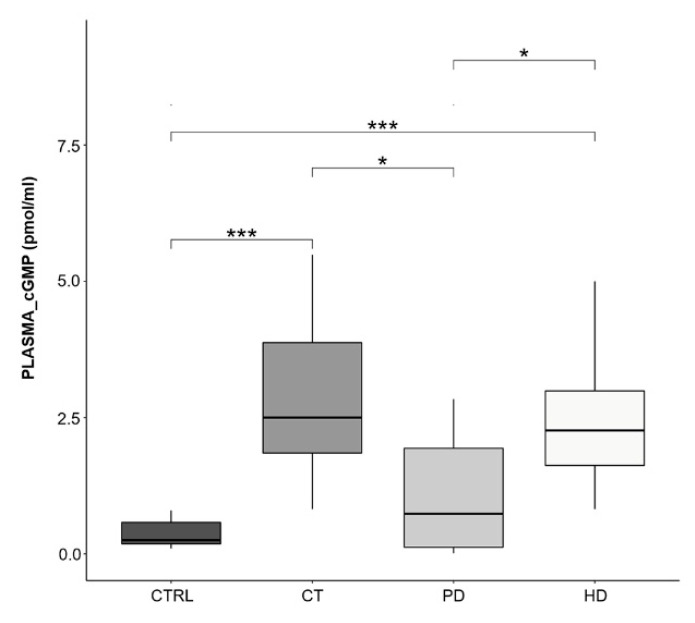
Plasma cGMP levels. Box plots represent plasma cGMP levels in the four study groups (CTRL, CT, PD, HD; *N* = 10 for each group). cGMP concentration was evaluated through ELISA and expressed as picomoles/milliliters (pmol/mL). Horizontal black lines indicate the medians, boxes the interquartile range (IQR) and the bottom and top whiskers represent the minimum and maximum values. (* *p* < 0.05; *** *p* < 0.001).

In agreement with these studies, we found that plasma cGMP content was significantly greater in plasma from CT and HD treated patients compared to both PD and CTRL subjects (Figure 5). Of note, levels in PD therapy patients were comparable to those from healthy subjects.

Based on what was postulated in the previous studies, it could be hypothesized that since PD is a continuous dialysis modality (day and night dialysis solutions) it would probably avoid the accumulation of cGMP [38].

### 2.7. Pre- and Post-Hemodialysis cGMP Levels in Plasma and RBCs from ESRD Patients

To test the hypothesis of plasma cGMP retention, we then evaluated potential changes both in plasma and RBCs cGMP levels before and after dialysis in a subgroup of ESRD patients on HD.

**Figure 6:**
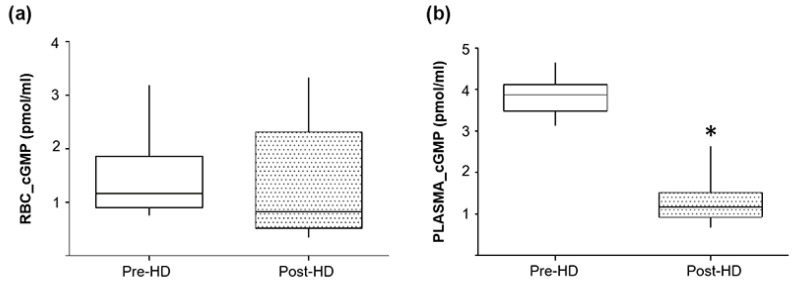
Pre- and post-hemodialysis cGMP levels in RBCs and plasma. Box plots representing (**a**) RBCs and (**b**) plasma cGMP levels in ESRD patients before and after hemodialysis (HD, *×* = 10). cGMP concentration was evaluated through ELISA and are expressed as picomoles/milliliters (pmol/mL). Horizontal black lines indicate the medians, boxes the interquartile range (IQR) and the bottom and top whiskers represent the minimum and maximum values. (* *p* < 0.05).

As shown in Figure 6b, the plasma cGMP level after hemodialysis was significantly reduced compared to the predialysis condition (1.37 ± 0.6 vs. 3.82 ± 0.5 pmol/mL, respectively). Thus, also in agreement with previous studies [15,37], our data allows us to confirm that the plasma accumulation of cGMP is possibly mainly caused by a reduced renal clearance.

On the other hand, as regards the content of cGMP in RBCs (Figure 6a), this was not reduced or otherwise altered following dialysis treatment, reinforcing the hypothesis that intra-erythrocyte accumulation is probably caused by the MRP4 altered transport activity.

### 2.8. Plasma Nitrite Levels

Plasma nitrite levels are reliably measurable and considered an indirect index of circulating NO levels. Moreover, their low levels have been suggested as an index of endothelial dysfunction related to cardiovascular risk factors [39]. Based on this, we finally measured the plasma nitrite levels in our study population.

**Figure 7:**
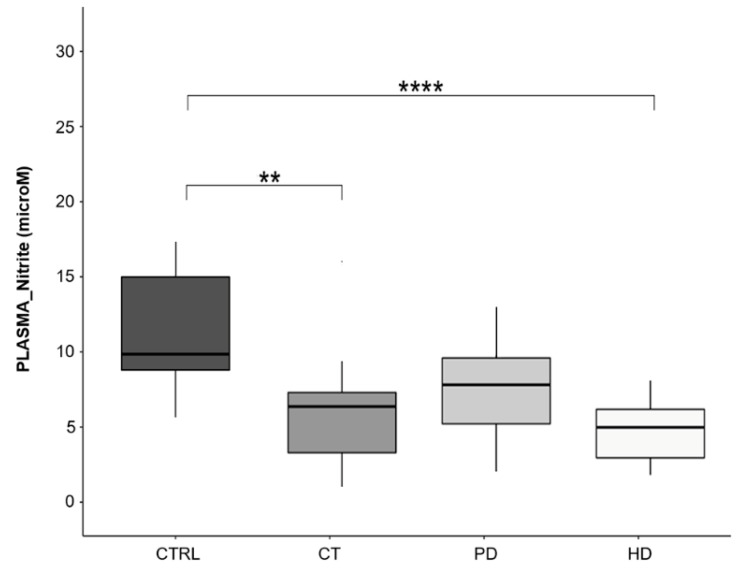
Plasma nitrite levels. Box plots represent plasma nitrite levels in the four study groups (CTRL, CT, PD, HD; *N* = 20 for each group). Nitrite concentration was determined spectrophotometrically and expressed as micromolar (microM). Horizontal black lines indicate the medians, boxes the interquartile range (IQR) and the bottom and top whiskers represent the minimum and maximum values. (** *p* < 0.01; **** *p* < 0.0001).

As shown in Figure 7, patients undergoing peritoneal dialysis showed plasma nitrite levels comparable to those found in healthy subjects, while they were significantly reduced in patients on CT and HD therapies (6.3 ± 2.5 and 5.9 ± 1.4 microM, respectively) compared to CTRL subjects (11.12 ± 4.5 microM).

Consequently, as for other parameters, the patients on PD therapy can be considered closer to the health condition, also as regards the greater circulating availability of nitrites.

## 3. Discussion

In the present study, our attention was mainly focused on the assessment of CKD-RBCs synthetic NO pathway that could be differently affected by the therapies used for the treatment of CKD, such as conservative (CT) and dialysis (PD and HD). Our data suggest that compared to PD, although eNOS activation in erythrocytes was similarly increased, CT and HD therapies were associated with an increased plasma and intra-RBC levels of cGMP, decreased MRP4 activity in RBCs and a parallel augmentation of IMT values associated to a reduction of plasma nitrite levels.

More specifically, RBCs from CKD patients on CT, PD and HD therapies showed comparable increase in eNOS phosphorylation levels than RBCs from healthy subjects (Figure 1). Although the processes responsible for increased RBC-NOS activation in erythrocytes from uremic patients have not yet been clearly defined, these results agree with our previous study that proved, besides a reduced level of eNOS expression, an increase of its activation in RBCs from hemodialyzed ESRD patients [8]. In this regard, several studies have shed light on a number of mechanisms able to activate eNOS to compensate the decreased NO plasma bioavailability in uremia [8,16,17,18]. Additionally, since it is conceivable that the change of intra-erythrocyte NO synthetic pathway could be influenced by several factors, such as patient’s disease history, severity, intervention regimens, and maintenance status, we enrolled patients looking for the best possible matching between groups in consideration of the parameters mentioned above.

Afterwards, in parallel to the increased eNOS activity, we observed that RBCs from CKD patients showed, compared to healthy ones, a significant increase of cGMP level, which represents the biological effector of the available NO. However, compared to erythrocytes from patients on CT and HD, the levels of this nucleotide were significantly lower in RBCs from patients on PD.

In general, the cGMP concentration in RBCs results from a balance between synthesis, through activation of soluble Guanylate Cyclase (sGC) by NO [40], and its removal. In particular, cGMP intra-RBC level could be affected by both hydrolysis, through phosphodiesterase (PDE) activity [41], and by its efflux via specific membrane proteins active transporters, such as MRP4 and MRP5 [21].

Concerning the identification of phosphodiesterase in human erythrocytes, to our knowledge it was based essentially on pharmacological studies, that is, PDE proteins are not easily identified, and it demands further investigation [42]. Thus, in the present study we focused attention on the MRP4 active transport instead of evaluating the phosphodiesterase potential role.

Among the factors potentially involved in the modulation of MRP4 activity, the increase in nitro-oxidative stress, possibly associated with the uremic condition, can be considered [43,44]. In particular, the nitro-oxidative stress consequent to the increase of harmful radical species, such as peroxynitrite [45], might induce in CKD-RBCs a reduction of NO bioavailability and then cGMP level. However, compared to other therapies, here we suppose that the PD treatment is associated to a lower RBC nitro-oxidative stress level since cGMP membrane transporter nitration and nitrosylation levels (NOTyr- and NOCys-MRP4) were significantly lower than those in CT and HD (Figure 3).

Considering that we have previously demonstrated that the MRP4 structural and functional changes led to a reduced efflux of cGMP and then accumulation within RBCs from hemodialysis subjects [8], here we hypothesized that this mechanism might be differently affected by the therapies employed for the treatment of CKD.

Thus, based on increased NOTyr- and NOCys-MRP4 levels found in RBCs from patients on CT and HD, we demonstrated that the ATPase activity of this membrane transporter was significantly decreased in these RBCs than those from patients on PD (Figure 4), justifying the intra-RBCs cGMP increase.

This could be explained by the fact that peritoneal dialysis allowing daily purification of the uremic environment could have favorable effects on nitro-oxidative stress as compared to conservative therapy and hemodialysis [38].

In parallel, also plasma cGMP level was increased by both CT and HD and it significantly decreased after hemodialysis. These data agree with other studies demonstrating an increase in plasma cGMP in CKD patients on hemodialysis therapy [15]. Interestingly, by measuring the pre- and post-dialysis cGMP levels in plasma from patients on HD, we found that its level significantly decreased post-dialysis (Figure 6), thus suggesting that this might be caused by a reduced renal clearance.

On the other hand, our data showed that plasma cGMP levels in patients on PD were comparable to those from healthy subjects, suggesting that this may be also due to a better preservation of PD residual kidney function compared with HD [46]. Moreover, on the basis of the present and previous studies, it could be assumed that as peritoneal dialysis is a continuous dialysis modality (day and night dialysis solutions) it would avoid the accumulation of cGMP (Figure 5).

Regarding the role of extracellular cGMP, although there is little evidence in the literature, it has been suggested a link between plasma cGMP and large artery remodeling in asymptomatic men. Especially, Devynck and colleagues verified a positive correlation between plasma cGMP levels with both high-sensitivity C-reactive protein concentration or multiple atherosclerotic plaques and IMT. This suggests a link between cGMP pathway, endothelial function and arterial wall geometry that is revealed by vascular injury conditions and may participate in early large artery remodeling [47].

As for the intra-erythrocyte cGMP levels, these were not influenced by the dialysis process, in fact they were similar to the levels measured pre-dialysis, confirming that the accumulation is mainly caused by a lack of efflux which is not improved post-dialysis.

Another interesting point that may contribute to better elucidate the complexity of NO bioavailability regulation in uremia, is the evaluation of plasma nitrite levels which represent a recognized indirect index of circulating NO levels. In this study, we show that PD therapy did not significantly influence nitrite levels as compared to the healthy condition, while CT and HD treatments showed significant reduction (Figure 7).

Although the evidence in the literature is rather small and conflicting, our data are in agreement with some studies that showed a reduced NO availability in plasma and urine samples from patients with CKD and also in murine models of uremia [48,49,50]. However, our results are in contrast with results showed by Thuraisingham and colleagues [51] that proved increased plasma nitrite and nitrate levels in HD patients, explained as an excess of NO production induced by the pro-oxidant uremic environment, although not bioactive.

Since it has been shown that plasma nitrite levels are reduced with endothelial dysfunction and their decrease is correlated with increasing number of cardiovascular risk factors [39], our data strengthen the idea that PD therapy might be less likely to develop endothelial dysfunction.

Of note, plasma nitrite levels were also found to positively correlated with flow-mediated dilation and inversely correlated with IMT [39].

In this regard, our results on carotid IMT values showed a non-significant increase in patients on PD therapy compared to healthy subjects, while it was significantly higher in CT and HD (Table 1). These data are consistent with previous studies, which demonstrated that carotid IMT was higher in CKD population compared with healthy subjects and well correlated with many cardiovascular risk factors [52]. Thus, although the small population analyzed avoids a correlation between IMT and plasma nitrite levels, it permits us to speculate that in a larger population a potential inversely correlation could be found. Furthermore, IMT values demonstrated in patients on PD allow us to assume that this therapy might less affecting vascular structures and functions [53,54].

All together our results demonstrated that compared to PD, both HD and CT were associated to a reduced activity of cGMP membrane transporter, RBCs-cGMP accumulation and parallel augmented plasma cGMP level, that significantly decreased after hemodialysis. Additionally, compared to healthy subjects, plasma nitrite levels were significantly reduced by HD and CT but not in patients on PD and notably the increased carotid intima-media thickness values did not reach the significance exclusively in patients on PD.

**Figure 8:**
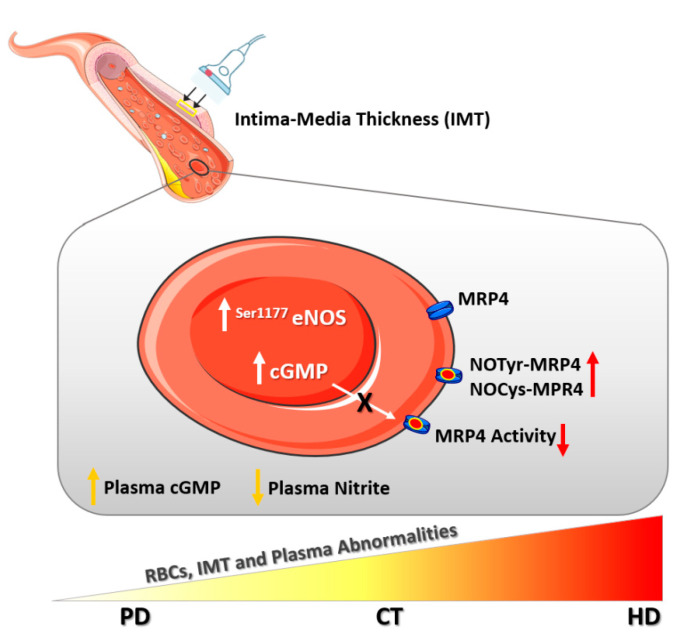
Red blood cell (RBC) and plasma abnormalities in Chronic Kidney Disease (CKD) progression. Uremic RBC showed a compensatory increase of eNOS (endothelial Nitric Oxide Synthase, also named RBC-NOS) phosphorylation levels along with intra-erythrocyte accumulation of cGMP (cyclic Guanosine Monophosphate). The latter mainly caused by decreased ATPase activity of MRP4 (multidrug-resistance-associated protein-4), a known cGMP membrane transporter, caused by rising levels of tyrosine nitration and cysteine nitrosylation of this transporter (NOTyr-MRP4 and NOCys-MRP4). Moreover, plasma levels of cGMP increased, potentially as a consequence of reduced renal clearance, while plasma Nitrite levels decreased. Notably, Peritoneal Dialysis (PD) impaired these parameters to a lesser extent than other CKD therapies. In parallel, the carotid Intima-Media Thickness (IMT), which was measured on the right and left common carotids, significantly increased in patients on Conservative Therapies (CT) and Hemodialysis (HD), while in patients on PD was comparable to healthy subjects.

In conclusion, our results show that PD might better preserve the synthetic NO-pathway in CKD-erythrocytes and then, taken in consideration also IMT values, it might be associated to a reduced development of uremic vascular complications (Figure 8), although at present this can only be hypothesized, and further clinical studies are clearly needed to confirm these preliminary observations.

However, our findings add a piece of knowledge that supports peritoneal dialysis in the rather controversial scenario of the effect of CKD therapies and allows us to ambitiously hypothesize that one or more RBC and/or plasma abnormalities found, if verified in a larger population, could develop into potential biomarkers for monitoring cardiovascular risk in uremia and possibly in other chronic diseases as well.

## 4. Materials and Methods

### 4.1. Study Population

In order to reach the main purpose of our study, 60 CKD patients were consecutively enrolled among those presenting at the Nephrology Division of Chieti University Hospital. Specifically, 20 CT (CKD stages 3–4), 20 PD (CKD stage 5, ESRD) and 20 HD (CKD stage 5, ESRD) were recruited along with 20 age- and gender-matched healthy subjects as control group (CTRL).

Subjects were considered eligible based on the following criteria: age between 18 and 80 years; CKD between third and fifth stage; absence of other erythrocyte-related diseases, diabetes mellitus, uncontrolled hypertension, infections, tumors, and inflammatory diseases, iron and folic acid deficiency, blood transfusion in the previous three months. All patients were on stabilized erythropoietin (EPO) dosage. In addition, subjects treated with drugs that might interfere with erythropoiesis (such as angiotensin-converting enzyme inhibitors and theophylline), NO synthesis or oxidative state were excluded.

Moreover, as shown in Table 1, several clinical and biochemical parameters were analyzed in the study population: body mass index (BMI), total cholesterol, high-density lipoprotein (HDL), low-density lipoprotein (LDL), and triglycerides as nutritional status indexes; creatinine as indexes of renal function; hemoglobin and hematocrit as indexes of the potential anemic state; C reactive protein (CRP) as inflammatory state indicator; other parameters such as glycemia, total proteins, systolic and diastolic blood pressure. In addition, carotid intima-media thickness (IMT) was assessed by B-mode ultrasound, as a marker of subclinical atherosclerosis. IMT data resulted from the average of 6 measurements performed on both right and left common carotids.

The study protocol was approved by the Ethics Committee of the University of Chieti, and, in adherence with the Declaration of Helsinki, written informed consent was obtained from all subjects taking part in the study.

### 4.2. Blood Collection

Venous blood samples from healthy subjects and CKD patients were collected into four sodium citrate vacuum tubes (4 mL each). For ESRD patients in hemodialysis venous withdrawals were performed before the treatment, while in a subgroup of 10 subjects blood samples were collected both before and after hemodialysis to evaluate its effect on RBCs and plasmatic cGMP content. Briefly, whole blood was used for flow cytometry analysis whereas for all other experimental protocols tubes were centrifuged at 500× *g* for 10 min at room temperature (RT) to isolate RBCs and plasma fractions and then differently treated for further investigation.

### 4.3. Materials and Antibodies

Phosphate-buffered saline (PBS), D-glucose, bovine serum albumin (BSA), sodium orthovanadate, sodium fluoride, sodium pyrophosphate, HEPES, sodium chloride, ethylenediaminetetraacetic acid (EDTA), leupeptin, aprotinin, phenylmethylsulphonyl fluoride (PMSF), N-ethylmaleimide (NEM), glutaraldehyde, anti-glycophorin A (CD235a BV421), anti-S-nitroso-cysteine and anti-band 3 were purchased from Sigma Aldrich (Saint Louis, MO, USA); Absolute Ethanol was form Carlo Erba (Milan, Italy); Triton—X100 was from ICN X100 Biomedicals (Eschwege, Germany); Hydrochloric acid was purchased from Titolchimica (Rome, Italy). Phospho-eNOS (Ser-S1177) was purchased from Aviva System Biology (San Diego, CA, USA); Anti-MRP4 was from Abcam (Cambridge, MA, USA); Anti-eNOS PE conjugated was from Miltenyi Biotec (Bologna, Italy); Anti-nitro-tyrosine was from Millipore (Billerica, MA, USA); Anti-rabbit Alexa 488 was from Thermo Fisher (Milan, Italia).

### 4.4. Flow Cytometry Analysis

The activation of the eNOS was evaluated in RBCs by flow cytometry analysis. Briefly, primary antibodies against eNOS and phospho-eNOS were incubated with fixed and permeabilized RBCs (with 0.05% Glutaraldehyde and 0.01% Triton X-100 respectively) for 30 min at 4 °C. Following a washing step with PBS without Ca^++^/Mg^++^, secondary antibodies were added for 30 min at 4 °C. Finally, the antibody against the constitutively erythrocytic membrane protein Glycophorin A (1:100) was used to specifically target RBCs.

Sample stained with the corresponding secondary antibody alone were also used as negative control. A total of 1 × 10^4^ events/samples were acquired by fluorescent-activated cell sorting (FACS) Canto II (BD Biosciences, San Diego, CA, USA). All data were analyzed using FACS Diva (BD Biosciences) and FlowJo v.8.8.6 software (TreeStar, Ashland, OR, USA) and expressed as mean fluorescence intensity (MFI) ratio. The MFI ratio was calculated by dividing the MFI of positive events by the MFI of negative events (MFI of secondary antibody).

### 4.5. Evaluation of cGMP Levels

To assess plasmatic and intra-erythrocytic cGMP levels, in a subgroup of 10 subjects of each condition (CTRL, CT, PD, HD) plasma and RCBs were processed to obtain suitable samples for the enzyme-linked immunosorbent assay (ELISA). Regarding the plasmatic fraction, it was treated with absolute ethanol whereas RBCs were washed thrice with a washing solution (Glucose 10 mM and BSA 10 mg/mL in PBS w/o Ca++/Mg++) to remove potential mononuclear cells residues and HCl 1N was then added. Both plasma and erythrocytes samples were successively dry-frozen. Then, 500 µL of plasma and 500 µL of RBCs were processed following the manufacturers’ instructions (Cyclic GMP ELISA Kit, Cayman Chemical Company, Ann Arbor, MI, USA).

The absorbance at 520 nm was read by using a microplate reader (SpectraMAX 190, Molecular Devices, Sunnyvale, CA, USA) and it was inversely proportional to the amount of cGMP contained in the analyzed samples. cGMP concentration was calculated using a standard curve as reference and data were expressed as pmol/mL.

### 4.6. Western Blot Analysis

MRP4 expression, nitration, and nitrosylation levels were evaluated in a subgroup of 10 subjects of each condition (CTRL, CT, PD, HD). Briefly, RBCs were washed twice with PBS w/o Ca^++^ and Mg^++^, once with the washing solution and conserved aliquoted in the lysis buffer (sodium orthovanadate 2 mM, sodium pyrophosphate 4 mM, sodium fluoride 10 mM, HEPES 50 mM pH 7.9, sodium chloride 100 mM, EDTA 10 mM pH 8, Triton 1%, leupeptin 2 µg/mL, aprotinin 2 µg/mL, PMSF 1 mM and distilled water) at −80 °C until analysis.

Then, RBCs were processed as previously described [8] (Di Pietro N et al., 2016). Briefly, 50 µg of RBC membrane proteins were separated by SDS-PAGE and incubated with anti- MRP4 (1:500) and anti-band 3 (1:5000, positive control) primary antibodies. Protein levels were then detected by enhanced chemiluminescence (Bio-Rad).

To evaluate the MRP4 nitration in tyrosine and nitrosylation in cysteine, 500 µg of membrane proteins were immunoprecipitated with primary antibodies against Nitro-tyrosine or S-nitroso-cysteine (both 1:100) with 20 uL of Pure Proteome protein A and protein G Magnetic Beads (Millipore), resolved by SDS-PAGE and probed with an anti-MRP4 antibody. The densitometric analysis of Western blots was performed using ImageJ software and data were expressed as arbitrary units (A.U.).

### 4.7. MRP4 ATPase Activity

To assess MRP4 ATPase activity, in the same subgroup considered for MRP4 determination, RBCs samples were processed as described in paragraph 4.8. Then, 500 μg of membrane proteins were immunoprecipitated with a primary antibody against MRP4 (1:100), as described by Di Pietro N et al. (2016) [8]. Concisely, the samples were incubated for 30 min at 37 °C with a reaction mix (25 mM Tris pH 7.0, 3 mM ATP, 50 mM KCl, 2.5 mM MgSO4, 3 mM dithiothreitol, 0.5 mM EGTA, 2 mM OUABAIN, 3 mM mol/L sodium azide) and incubated for 30 min at RT with an ice-cold stopping buffer (0.2% *w*/*v* ammonium molybdate, 1.3% *v*/*v* H2SO4, 0.9% *w*/*v* SDS, 2.3% *w*/*v* trichloroacetic acid, 1% *w*/*v* ascorbic acid). Then, the absorbance at 620 nm was measured using the microplate reader Packard EL340 (Bio-Tek Instruments, Winooski, MA, USA). The results were expressed as MRP4 ATPase activity %.

### 4.8. Assessment of Plasma Nitrite Levels

Since nitrites are one of the primary stable and nonvolatile breakdown products of NO, we evaluated plasmatic nitrite levels through the Griess assay. Briefly, 100 μL of plasma obtained as described in paragraph 4.2 were processed following the manufacturer’s instructions (Griess Reagent System, Promega, Fitchburg, WI, USA). Nitrite’s concentration was determined by the spectrophotometric absorbance measurement at 520 nm (SpectraMAX 190, Molecular Devices, Sunnyvale, CA, USA) and expressed as micromoles (mM).

### 4.9. Statistical Analysis

The data were processed using the R statistical software (version 3.5.0). Both parametric (one-way analysis of variance, ANOVA) and non-parametric (Kruskal-Wallis) tests were used to analyze our results.

For each parameter analyzed, a first comparison was made between the mean of all 4 groups (CTRL, CT, PD, HD), followed by a second comparison in pairs (i.e., CTRL-PD, PD-HD).

Differences were analyzed using the Bonferroni post hoc test for the parametric data and the corresponding Dunn’s test for the non-parametric data.

Data distribution was represented in box plots, median was used as a measure of central location and significance are reported as *p* < 0.05. The differences in significance were indicated using a different number of asterisks on top of box plots.

## Figures and Tables

**Table 1:** Main clinical and biochemical characteristics of the study population.

		CKD			
	CTRL (*N* = 20)	CT (*N* = 20)	PD (*N* = 20)	HD (*N* = 20)	*p*-Value
Gender (female/male)	10/10	10/10	6/14	9/11	
Age	53 ± 11	64 ± 16	56 ± 22	65 ± 15	ns
BMI	24.8 ± 4.2	24.5 ± 4.1	26.9 ± 4.7	26.1 ± 5.9	ns
Systolic Blood Pressure (mmHg)	113 ± 11.2	138 ± 18.9 *	140 ± 20.8 *	127 ± 21.8 *	˂0.05
Diastolic Blood Pressure (mmHg)	71 ± 8.4	76 ± 11	78 ± 10	72 ± 14	ns
IMT	0.64 ± 0.1	0.95 ± 0.3 *	0.86 ± 0.3	0.99 ± 0.2 *	˂0.05
Hematocrit (%)	43.6 ± 4.3	36 ± 4.9 *	35.7 ± 3 *	34.8 ± 4.1 *	<0.05
Hemoglobin (g/dL)	14.4 ± 1.5	11.4 ± 1.6 *	11.3 ± 1 *	10.7 ± 1.2 *	˂0.05
Creatinine (mg/dL)	0.83 ± 0.2	4.08 ± 3 *	9.93 ± 4 * ^§^	8.37 ± 2 * ^§^	˂0.005
Albumin (g/dL)	4.54 ± 0.51	3.98 ± 0.58 * ^#^	3.49 ± 0.40 *	3.89 ± 0.45 * ^#^	˂0.05
Total Proteins (g/dL)	7.57 ± 0.55	6.89 ± 0.94 * ^#^	6.48 ± 0.66 *	6.59 ± 0.55 *	˂0.05
Glycemia (mg/dL)	88.8 ± 13.2	82.7 ± 15.4	93.2 ± 34.6	91.4 ± 35.2	ns
Triglycerides (mg/dL)	127.1 ± 49	132.8 ± 62 *	144.1 ± 69 *	154.1 ± 85 *	˂0.05
Total Cholesterol (mg/dL)	167.8 ± 42.7	168.8 ± 51.9	152.2 ± 37.8	143.8 ± 31.9	ns
Cholesterol HDL (mg/dL)	55.5 ± 21.1	52.3 ± 16.9	43.8 ± 13.4	48.7 ± 22.9	ns
Cholesterol LDL (mg/dL)	88.7 ± 36.9	90.2 ± 39.4	77.3 ± 27.8	64.3 ± 21.6	ns
CRP (mg/dL)	0.30 ± 0.12	0.70 ± 0.75 *	0.80 ± 1.03 *	1.12 ± 1.60 *	˂0.05

Data are mean ± SD. BMI, Body Mass Index; IMT, Intima Media Thickness; HDL, High-Density Lipoprotein; LDL, Low-Density Lipoprotein; CRP, C Reactive Protein. * *p* < 0.05 vs. CTRL; ^#^ *p* < 0.05 vs. PD; ^§^ *p* < 0.005 vs. CT.
